# A systematic review of measures of healthcare workers’ vaccine confidence

**DOI:** 10.1080/21645515.2024.2322796

**Published:** 2024-03-20

**Authors:** Kofoworola O. Akinsola, Ayobami A. Bakare, Elisa Gobbo, Carina King, Claudia Hanson, Adegoke Falade, Sibylle Herzig van Wees

**Affiliations:** a Department of Pediatrics, University College Hospital, Ibadan, Nigeria; b Department of Community Medicine, University College Hospital, Ibadan, Nigeria; c Global Public Health Department, Karolinska Institutet, Stockholm, Sweden; d Global Public Health Department, Karolinska Institute, Stockholm, Sweden; e Department of Disease Control, London School of Hygiene & Tropical Medicine, London, UK; f Centre of Excellence for Women and Child Health, Aga Khan University, Nairobi, Kenya; g Pediatrics Department, University of Ibadan, Ibadan, Nigeria

**Keywords:** Healthcare workers, vaccine confidence, validation, survey tools, hesitancy, systematic review

## Abstract

Healthcare workers (HCW) perceptions toward vaccines influence patient and community vaccine decision making. In an era of rising vaccine hesitancy, understanding HCW vaccine confidence is critical. This systematic review aims to review instruments that have been validated to measure HCW vaccine confidence. We conducted a search in five databases in June 2023. Data was descriptively synthesized. Twelve articles describing 10 different tools were included. Most tools included dimensions or items on vaccine knowledge (*n* = 9), safety (*n* = 8), vaccine usefulness (*n* = 8), recommendation behavior (*n* = 8), and self-vaccination practice (*n* = 7). All, except one study, were conducted in high-income countries. There was variability in the quality of the validation process. There is limited existing literature on development and validation of tools for HCW vaccine confidence. Based on the tools currently available, the Pro-VC-Be tool is the most well validated. Further research needs to include low- and middle-income contexts.

## Introduction

Vaccination plays a pivotal role in public health by reducing morbidity and mortality associated with infectious diseases and their acute and long-term manifestations.^1^ Between 2010 and 2018, measles vaccination alone has prevented over 20 million deaths.^2^ Among the key drivers behind vaccination success are healthcare workers (HCWs), who serve as front-line providers of immunization services.^3^ There is substantive evidence to suggest that HCWs are the most trusted advisors and influencers of vaccine decision making among patients.^4–8^ Some evidence suggested that low confidence in vaccines among HCWs has been associated with low uptake rates of the vaccines.^9,10^

Vaccine hesitancy is a complex phenomenon. It has been defined as a behavior in delaying or refusing a particular vaccine or vaccination generally despite availability,^11^ or as a state of indecisiveness regarding vaccination.^12,13^ Vaccine hesitancy has been observed throughout the world in the past decade,^14^ however, since the introduction of the COVID-19 vaccine, vaccine hesitancy has increased globally, including among HCWs.^15–18^ For example, the coverage of diphtheria-tetanus-pertussis (DTPcv1) containing vaccines decreased by 21% between 2021 and 2022, and one of the factors impacting the drop in coverage is vaccine hesitancy.^19^

A Strategic Advisory Group of Experts on Immunization working group on vaccine hesitancy proposed the 3Cs of vaccine hesitancy as confidence (trust in vaccines and those delivering them), complacency (low risk, not necessary to take preventative measures), and convenience (affordability and accessibility).^11^ Terminology around vaccine hesitancy and confidence is not consistent throughout the literature, and we refer to vaccine confidence throughout this paper. In an era marked by evolving vaccine production, increased public scrutiny, and rising vaccine hesitancy, it is imperative to gain a nuanced understanding of waning vaccine confidence among HCWs.^20^

The validation of survey tools or instruments is an essential component in measuring vaccine confidence among HCWs. Validation promotes reliable, accurate, and effective tools for measuring the complex constructs of vaccine behaviors among this important population.^21,22^ The process often involves rigorous statistical and methodological evaluation, as well as cross-cultural adaptations to ensure the tools’ appropriateness in various contexts.^21^ Tool validation is therefore important to ensure that the data collected reflect the unique characteristics and dynamics of HCW vaccine confidence.

There has been an increase in literature on HCW’s vaccine confidence using a variety of qualitative and quantitative methods. Examining the HCW’s vaccine confidence is of particular concern due to HCWs higher risk of transmission of illness,^14^ how their vaccine confidence influences their likelihood to recommend vaccines,^23^ and since HCWs are trusted sources for vaccine uptake among patients.^7,8^ These factors make it important to examine HCW vaccine confidence separately from parental or general population vaccine confidence.^23^ However, to our knowledge, there is currently no published systematic reviews on the validation of tools used to assess vaccine confidence among HCWs. Therefore, this systematic review aims to review the survey tools/instruments that have been developed and validated to measure HCW vaccine confidence. This review will provide valuable insights into the development of standardized and robust assessment measures of vaccine confidence among HCWs, which can inform more targeted interventions and policies aimed at enhancing vaccine confidence among HCWs.

## Methods

This is a systematic review. The review is based on current best practices utilizing the Joanna Briggs Institute (JBI) systematic review framework.^24^ The population, concept, context (PCC) framework was used to guide the development of our research question.^25^ The population being HCWs; the concept tools to measure vaccine hesitancy or vaccine confidence/trust, vaccine acceptance; context including a global setting. This framework as well as the literature review culminated in the research questions: What validated tools exist to measure healthcare worker vaccine confidence/hesitancy/acceptance? No review protocol exists, and the systematic review has not been registered.

### Search strategy

The PRISMA checklist and flow diagram were used to guide the search and presentation of results.^26^ The search strategy was developed in Medline (Ovid) in collaboration with librarians at the Karolinska Institutet University Library. Medline, Web of Science, CABI: CAB Abstracts, and Global Health and Sociological Abstracts were searched, and Publicly Available Content database was used as complementary search. The last search was conducted in 2023-06-08. For each search concept Medical Subject Headings (MeSH-terms) and free text terms were identified on Medline. No language restriction was applied, and databases were searched from inception with no date restrictions. The search was then translated, in part using Polyglot Search Translator.^27^ The strategies were peer reviewed by another librarian prior to execution. Some of the key search terms used were immunization, immunization programs, exp vaccination, exp vaccines, vaccine confidence, vaccine hesitancy, vaccine acceptance, anxiety, awareness, behavior, choice behavior, communication barriers, health knowledge, attitude, and practice, intention, health personnel, benchmarking, health care surveys, quality assurance, health care, survey and questionnaire. The full search strategy is available in Appendix A.

After running the searches in all databases, a de-publication process was done using the method described by Bramer et al.^28^ Finally, DOIs were compared to avoid duplicate articles. For the full search strategy, any articles that assessed vaccine confidence among HCW were included, then the research team narrowed to tool development and validation during the screening process.

### Eligibility criteria

We included articles that focused on measuring vaccine hesitancy/confidence/acceptance, vaccine behavior, or vaccine attitudes of HCWs. Specifically, we included peer-reviewed articles describing the tool development and validation process. The term HCWs referred to any group working with healthcare.^29^ The articles were included if the entire target population for the validation process was focused on healthcare workers or healthcare professionals. We included healthcare students in this definition of HCWs. We excluded systematic review or an intervention study.

### Selection process

We used Rayyan.ai software^30^ for the screening process. After the de-duplication, one researcher (EG) screened article titles and abstracts to create a short list. Initial inclusions were articles that measure healthcare worker vaccine confidence/hesitancy/acceptance that discuss how it was measured or what tools were utilized.

Two researchers (EG and KA) did a blinded title and abstract screening, which included only articles measuring tool validation with the focus of the studies’ results and discussions on how vaccine confidence was measured. From there, the articles were re-blinded and then full articles were screened using the eligibility criteria by EG and KA. Any discrepancies were discussed between EG, KA and senior researchers from the team (SHvW and BA) to determine final inclusion. Ten articles were included for the data extraction and quality assessment.^25^

### Data extraction and quality assessment

The extraction and quality assessment were done, blinded, by EG and KA using an extraction form developed by the team. The form was developed using guidance from JBI Framework.^24^

The QAVALS (Quality Appraisal Tool Specifically for Validation Studies) measure for instrument validation was utilized due to the focus on validation methods (Appendix B).^22^ The tool had yes, no, or other for each of 24 items on study design and validation methods. The tool items assessed articles study designs, selection criteria, testing procedure, statistical analyses, errors, reporting, face and content validity, criterion validity, and construct validity (known groups, convergent, discriminant). Based on the questions provided and the QAVALS item description guide, the researchers assessed each article as including the item (yes), not including the item (no), or as cannot be determined, not applicable, not reported (other). In the end, based on the quality assessment alone, no articles were excluded from this process. Any disparity in the overall assessment was mediated through discussion.

### Data synthesis and analysis

The extraction table was modified for clarity for the tabulation of results. Descriptive statistics were used for year of publication, country income level of study location, type of tool validated, healthcare worker type, vaccine studied, sample size, and topics/dimension assessed with the tool. A heat map was generated to examine the quality of the validation processes based on the QAVALS quality assessment tool.^22^ Based on the ratings given by EG and KA, each article was assigned a value of 1, 0.5, or 0 based on the 24 items in the assessment tool. Additionally, we added columns to say if the study included face and content validity, criterion validity, construct validity (known group, convergent, and discriminant), and reliability. A value of 1, shown in green on the map, was rated as yes, 0 (red) indicates no (item not included) and 0.5 (light green) indicates others.

## Results

**Figure 1.**
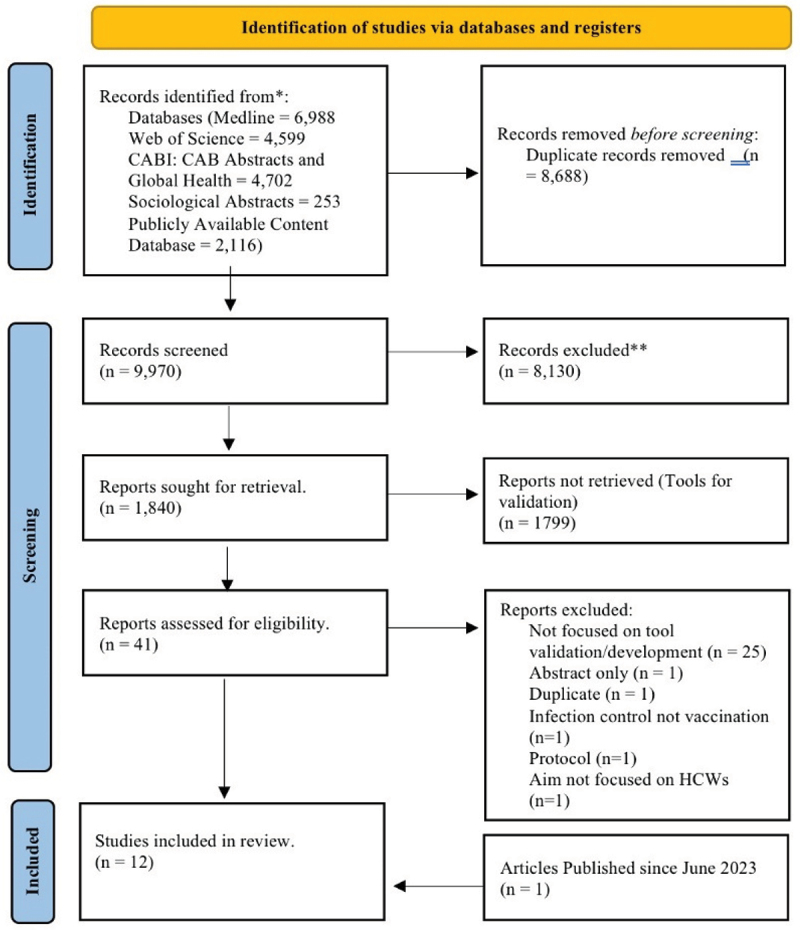
PRISMA flow diagram of article inclusion^26^.

Overall, 9970 articles were returned from the search, and 12 were finally included (Figure 1). The most common reason for article exclusion at full text review were: not focusing on tool development/validation,^25^ being a duplicate(1), being an abstract only(1), focusing on infection control rather than vaccination(1), aim not including HCWs(1), being a protocol(1).^31,32^ One additional article was added that was published after the last search but fit all inclusion criteria.^33^

Table 1 summarizes the aims, tools assessed, vaccines, dimensions of confidence measured by the tools, and results of the ten included articles. The majority of the articles (10/12) were published since 2013,^33-42^ and all expect one study were conducted in high-income countries.^33-41,43,44^ One study was in Malaysia, an upper middle-income country.^42^ Slightly over half (6/12) of the studies examined all vaccines,^33,35,36,41–43^ four focused on influenza,^34,38,39,44^ one focused on COVID,^40^ and one on HPV.^37^ The majority (9/12) included physicians, seven included nurses,^34,36,38,40–42^ five included general practitioners,^33,36,41–43^ three included healthcare students,^35,39,43^ two included all healthcare staff (including admin),^34,39^ and one included pharmacist^34^ as their target population during validation.

**Table 1.** Article summary information: title, authors, year of publication, study setting location, study aim, tool assessed, population type, sample size, and type of vaccine.

Authors	Location	Study Aim	Tool Assessed	Population	Sample Size	Vaccine
Fernandez-Prada et al^34,35^	Spain	Design the Questionnaire on the Attitudes of Healthcare Professionals towards the Official Flu Vaccination Recommendations (CAPSVA)	CAPSVA	Healthcare Professionals (nursing, medicine, and pharmacy) at Principado de Asturias	288	Influenza
Fernandez-Prada et al^35^	Spain	Design and validate a questionnaire that allows for the exploration of attitudes and behaviors of medical and nursing students in regard to vaccine and immune-preventable diseases during clinical practices.	ACVECS (Questionnaire on the Attitudes and Behaviors towards Vaccination among Health Sciences Students)	Health science students	646	All Vaccines
Garrison et al ^33^	France, Belgium, Canada	The objective of this study was to assess the construct and criterion validity of short for Pro-VC-Be with 10 items representing each of the10 dimensions from the long-form tool. And to generate a global score to measure immunization resourcefulness.	Short form of Pro-VC-Be (Health Professionals Vaccine Confidence and Behavior)	Healthcare Professionals (GPs, Physicians, nurses)	2,696	All Vaccines
Garrison et al^36^	Germany, Finland, France and Portugal	Purpose was to adapt and validate long- and short- form version of International Pro-VC-Be to measure psychosocial determinants of HCP’s Vaccine confidence and associations with vaccination behaviors across European countries	I-Pro-VC-Be International Health Professionals Vaccine Confidence and Behavior)	HCP (mainly GPs and pediatricians)	2,748	All Vaccines
Khamisy-Farah et al^37^	Israel	To investigate knowledge of HPV and HPV related issues, and attitudes and practices towards recommending HPV vaccine. KAP questionnaire developed and validated in a sample	KAP questionnaire	Pediatricians, gynecologists, and internal med docs	139	HPV
Paoli et al^38^	Italy	Access the phenomenon of vaccine hesitancy among HCWs by measuring it via a scoring system	Healthcare workers vaccination compliance index (HVCI)	Healthcare workers in pediatric hospitals (docs, nurses, assistants, lab techs)	108	Influenza
Prislin et al^43^	USA	Aimed at improving vaccination rates by targeting physicians, and developed measures of factors influence physician immunization practices and examine the reliability and validity of their measures.	Self-developed tool (Measures include knowledge, attitudes, vested interest, self-efficacy, and perceived barriers)	Physicians (residents, fam medicine, pediatric, primary care, and specialists)	209	All Vaccines
Slaunwhite^39^	Canada	3 studies in the dissertation. Focusing on Study 3. Tested the adequacy of the Theory of Planned behavior framework in explaining intention to receive vaccine including a modified Perceived Behavioral Control item set and tested the predicative ability of intention to receive vaccine on actual uptake.	Theory of Planned behavior framework with Perceived Behavioral Control	Healthcare workers from pediatric clinic (staff, physicians, students, and volunteers)	262	Influenza
Tomietto et al^40^	Italy	Aimed to validate the Italian version of the VAX scale and describe nurses’ attitudes towards COVID-19 vaccination	Vaccination Attitudes Examination (VAX)	Nurses	430	COVID
Verger et al^41^	France, Belgium, Canada	Aim to validate the Professionals Vaccine Confidence and Behaviors instrument. The instruments’ objective is to measure various psychosocial factors that may play a role in vaccine confidence and vaccine behavior among different types of HCPs.	Pro-VC-Be (Health Professionals Vaccine Confidence and Behavior)	GPs and Nurses	2736	All Vaccines
Kadir et al^42^	Malaysia	Aims to develop and validate knowledge and attitude regarding childhood vaccination (KACV) questionnaire among healthcare workers.	KACV	All HCWs	114	Childhood vaccines
Zhang et al^44^	United Kingdom	To develop an instrument to measure nurses’ knowledge, risk perceptions, and health behaviors towards influenza, influenza vaccine, and vaccine behaviors.	King’s Nurses’ Influenza Vaccination Questionnaire	Nurses	520	Influenza

**Figure 2.**
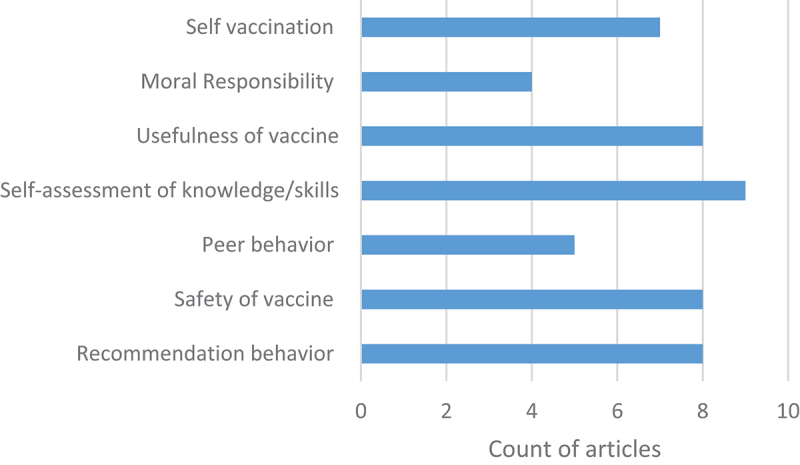
Topics covered by dimensions or items in tools presented in included articles (*n* =11).

Nine of the studies developed and validated novel tools.^34,35,37–39,41–44^ One study was based on the Vaccine Attitudes Examination (VAX) tool,^40^ which has been previously used for patient vaccine hesitancy, and two studies validated variations of the Health Professionals Vaccine Confidence and Behavior (Pro-VC-Be) tool.^33,36^ The tools examined different dimensions (illustrated in Figure 2) but covered many related topics. Nine of the twelve included studies assessed self-assessment of knowledge/skills of vaccination.^33,34,36,38,41–45^ Eight include items relating to recommendation behavior,^33–35,37,38,41^ safety of vaccines,^33,35–38,41–43^ and usefulness of vaccines.^33,35,36,38–41^ Seven tools gathered HCW perceptions on risk of vaccines,^33,36,38–41^ six gathered self-vaccination practices,^33,35,36,38,39,41^ five included influence of peer behavior^34,35,39,42^ and four gathered HCW’s sense of moral responsibility.^35,36,39,42,45^

The results from the studies mostly presented valid and reliable tools, but not all of the results focused on the validation process. Among the nine studies that reported Cronbach alpha scores for reliability, they all had a final score of above 0.70 on the different dimensions (Table 2). In four of the articles, they discussed that some items needed to be removed and adapted from the survey to have a strong reliability score.^33,39,41,43^ Validity coefficients were presented in the four studies that conducted a criterion validity, and all found good criterion validity among finalized version.^33,36,39,41^ Half the studies’ results and discussions primary focus were the results of the questionnaire itself rather than the validation process.^34,35,37,38,40,43^ Out of these six studies, four found that HCWs knowledge level is linked with their behavior or attitudes about vaccines.^34,35,37,38^

**Table 2.** Article dimensions, results, validation, and limitations.

Authors	Dimensions Studied	Results Summary	Cronbach alpha	Validation Obtained	Limitations
Fernandez-Prada et al^34,35^	Questions regarding opinions on new regional strategy. Dimensions include: characteristics of vaccines and training, influence of peers on recommendations, Sensitivity to awards and sanctions for vaccination.	Kaiser-Meyer-Olkin index was 0.90. 2 factors that explained 48.8% of total variance. The correlation factor was *r*=0.649. Overall knowledge score was 7.11 out of 10. Ordinal alpha value (internal consistency) for the tool 0.92. Factor most influenced by training time was knowledge.	0.92	Obtained conceptual and methodological rigor with internal validity and reliability.	Results may not be extrapolate to national level because not randomized sampling and not general representation of universities.
Fernandez-Prada et al^35^	Characteristics of the vaccines and trainings, influence of equals and organization on professional behavior, sensitivity to awards or sanctions for vaccine recommending	The standard deviation and item total correlation were adequate in all measures. 3-factor solution explained 79.38% pf total variance. A goodness of fit index of 0.99 was obtained. No difference by sex, doctor profile, but there was one between those with more or less than 5 years experience. No different based on having been vaccinated, or adverse events. Coverage of 70%.	N/A	Adequate indicators of internal validity and reliability found. Content validity, reliability, and internal validity	Self-repots carriers implicit bias. Low response rate. Limited to singular community in Spain. Sample size was adequate for instrument validation.
Garrison et al^33^	Perceived risks of vaccines, complacency, perceived benefit/risk balance, perceived collective responsibility, trust in authorities, perceived constraints, openness to patients, commitment to vaccination, self-efficacy, reluctant trust	Distribution of items varied between the countries. The configural invariance model fit the data showing that the factor structure was equivalent across groups. 8 of the 10 items had good convergent validity with all loadings >0.62 or >0.71. For openness to patients and perceived constraints in was fair (0.43 to 0.76). Discriminant validity varied across countries with France, Finland, and Portugal with weak to moderate correlations (good discriminate validity). But in Germany the correlations were higher. Criterion validity the Poisson regression showed that HCPs with higher scores of safter, benefit risk, collective responsibility, commitment to vaccination, self-efficacy, and trust in authorities were more like to recommend vaccines systematically (>90%).	Between 0.72 to 0.99 (except for openness to patients 0.37)	Cognitive validation, construct validity, and criterion validity.	Potential differences in vocabulary due to translation. Not validated in Non-western population. Self-reported bias (desirability bias)
Garrison et al^34^	Perceived risks of vaccines, complacency, perceived benefit/risk balance, perceived collective responsibility, trust in authorities, perceived constraints, openness to patients, commitment to vaccination, self-efficacy, reluctant trust	From each dimension found the item that was included in the majority of combinations with good to excellent fit. CFA model for short-form construct validity showed good fit. Items in confidence in vaccines had fair/good convergent and items in proactive efficacy had good/excellent convergent. Confidence in vaccines was moderately correlated with trust in authorities, all other dimensions were poorly or not correlated (similar to long form).Criterion validity was not the same for trust in authorities, reluctant trust, and perceived constraints.	0.71	Found construct validity and criterion validity, construct	Used attitudes towards COVID-19 for criterion validation, rather than a different indicator of actual vaccine behavior. Temporal restraints to taking country specifics of COVID vaccine roll out and country recs. Only high income and French speaking contexts.
Khamisy-Farah et al^35^	^1^ Knowledge of HPV and HPV-related burden,^2^ attitudes and practices towards HPV vaccination, and ^3^ awareness of safety and efficacy of vaccine with attitude	Gathered the Cronbach alpha for each of the predictor measures. Principle component analysis preformed on additional items to confirm factor structure. They did not find differences in terms of knowledge between residents or other doctor specializations. Only 20% did not recommend to boys.	0.74 and 0.85	Confirmed validity of the questionnaire with good internal consistency. KAP questionnaire found to be psychometrically valid and reliable	Small sample size and cross-sectional study design.
Paoli et al^36^	General info, self assessment on expertise, attitude towards flu vaccination and motivation, confidence, compliance, and risk perception	HCVI was statistically verified as a predictive parameter. 17% considered themselves to be poorly competent in understanding vaccines. Statistically significant differences between departments and professional profiles. 80% of population not vaccinated against flu.	N/A	Statistically verified	Only 31% of possible population completed the survey. HVCI is only is only one possible predictor of flu vaccination, others are strategic training policies and perceived skills.
Prislin et al^37^	Knowledge, attitudes, vested interest, perceived barriers	Overall response rate of 65%. Individual knowledge scale computed as a sum of all correct answers, and other scale as averages across the items on the scale. Alpha index for each scale: Knowledge (0.71), Vested interest (0.81), Self-efficacy (0.70), Attitudes (0.97), Perceived barriers (0.89). Construct validity indices separated by GPs, specialists, 3rd year residents, 1st year residents. Ranging from 1.82 to 9.37 as mean for generalists perceived barriers score.	Knowledge (0.71), Vested interest (0.81), Self-efficacy (0.70), Attitudes (0.97), Perceived barriers (0.89).	All scales prove internal consistency. Satisfactory construct validity (exp. Attitude scale)	Lacking generalizability, some social desirability bias
Slaunwhite^38^	Importance of flu shoot, consequences of not, ill from receiving flu shot (risks), individual choice, moral responsibility, % of employees with flu shot, opinion on flu shot, benefits and barriers intention to vaccinate	Cronbach alpha scores: Attitudes (0.92), perceived behavioral controls (0.51 and two item correlation 0.46), Descriptive normative influences (bivariate correlation of 0.36. Past behavior 216 reported receiving the seasonal influenzas vaccine the previous year. Outcome variables were intention to receive influenza vaccine with a mean score of 4.69, and behavior (vaccine uptake) which was 225 according to the data base the previous year. Addition of TPB variables accounted for significant increase in variance in behavioral intentions to receive the vaccine.	Attitudes (0.92), perceived behavioral controls (0.51 and two item correlation 0.46), Descriptive normative influences (bivariate correlation of 0.36.	Has good predictive validity	Internal consistencies for PBC sub scales were low (control sub scale Ca=0.46 and accessibility Ca=0.51). Possibly leads to inadequacy of TPB to predict intention.
Tomietto et al^39^	mistrust of vaccine benefit, worries about unforeseen future effects, concerns about commercial profiteering, preference for natural immunity	Overall mean value for the VAX scale score was 2.93. Highest mean score was detected in the “worries about unforeseen future effects” and lowest score was “mistrust of vaccine benefit.” Internal consistency was 0.89 and Cronbach alpha ranged from 0.77 to 0.86, the values did not increase with one by one deletion of items. EFA had a 76.3% variance. CFA using the ADF approach, with the 4-factors model verified by fit (RMSEA=0.045, SRMR=0.349,TLI0.868, CFI=0.908)	0.77 to 0.86	Content validity, reliability, construct validity	Benefit from larger sample to get a more normal model distribution for validation. Limited generalizability. Social desirability and auto-selection bias.
Verger et al^40^	Perceived risks of vaccines, complacency, perceived benefit/risk balance, perceived collective responsibility, trust in authorities, perceived constraints, openness to patients, commitment to vaccination, self-efficacy, reluctant trust	6-factor structure with good fit. EFA found nine factors with values >1 but did 10-factor solution for closer fit to theoretical constructs. CFA confirmed the 10-factor structure. Found moderate correlation between perceived risks of vaccines, perceived benefit-risk balance, complacency, and perceived collective responsibility and made them into a second order factor of vaccine confidence. Construct validity: first order and second order factors all had good (>0.63) to excellent (>0.71) convergent validity. Criterion validity: Poisson regression adjusted for age and gender. Probability of very frequent recommendations was 40% higher for GPs with above average vaccine confidence scores and it was similar for nurses.	Removed some items that lowered Cronbach alpha. Between 0.35 to 0.78	Good convergent and criterion validity and adequate discriminant validity. Further validation happened later in other languages.	Test-retest not yet completed. Could not measure convergent and divergent validity against other instruments because none yet validated for HCW vaccination behavior. Does not include knowledge. Only validated in French.
Kadir et al^41^	Knowledge section (advantages of vaccination, side effects/adverse reactions, methods/sites/types of vaccination, and myths. Attitude section was unidimensional about disease severity, disease susceptibility, efficacy, safety, key immunization beliefs, social influences, and main source of information.	Content validity: ICV for both domains is 0.92. 10 items were removed and 1 was added. Psychometric analysis: 6 items removed from knowledge section due to the ceiling effect. Validation and reliability: sphericity tests were significant enough that the data was suitable for further analysis. But Domain B had a Cronbach alpha below 0.7, so that was removed. Cronbach alpha for final questionnaire was 0.896 for knowledge, and 0.763 for attitude.	0.896 (Knowledge) and 0.763 (attitude)	Content validity, face validity, and construct validity, and reliability	Developed in Malay and only be used by the population that understand this language. The results showed a lack of variability across the Likert scale items (6 items removed due to ceiling effect). 90% of responses had correct answers for the knowledge section, which suggests the questions were easy. Validation done in a tertiary center.
Zhang et al^42^	Knowledge about influenza and the vaccine, perception of risk of influenza, health beliefs, practices regarding vaccination, reasons for or against having the influenza vaccine	Expert discussions resulted in adjustment of wording of items, and two open items being replaced with closed ones. From the pilot study formatting was changed to make it look shorter. Cronbach alpha coefficients ranges from 0.701 to 0.763 for each scale and fit internal consistency criteria. EFA of seasonal influenza knowledge with nine factors. One item was removed due to low factor loading.	0.701 to 0.763	Found good construct validity and internal consistency reliability.	Test results done with a convenience sample, knowledge scores of participants were quite low, and done to measure nurses in the UK so not generalizable.

### Quality of tool validation

The heat map shows that the quality of the validation processes was mixed across the ten studies. Generally, the study design was well described and appropriate for a tool validation study. Yet, six of the articles did not report a sample size calculation.^35,37–39,41,43^ The study sample sizes had a median of 359, but a range of 108 to 2748 participants.

**Figure 3.**
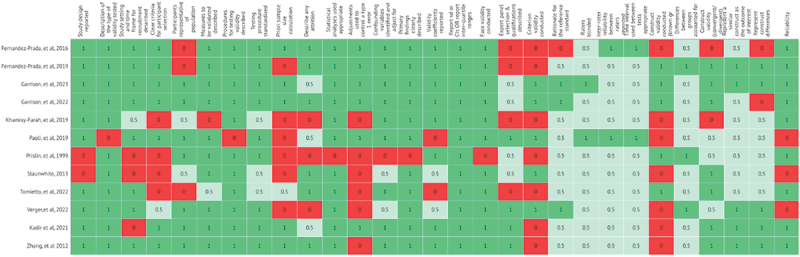
Heat map of article quality assessment and validation Process.^46^.

There was also variability in the type of validations conducted. Three studies did all three main forms of validation: face validity, criterion validity, and construct validity.^33,36,41^ Among the 12 studies that examined face validity, they generally were of good quality, and in Kadir et al. they conducted both a content and face validity separately.^42^ As the red and light green on the heat map demonstrate, criterion and construct validity were either not conducted or of a medium quality. Four studies conducted a criterion validity utilizing vaccination behavior as the comparison,^33,38,39,41^ and one used the long-form Pro-VC-Be tool as the criterion for the short-form version.^36^ None of the other studies gave a rationale for why they did not conduct a criterion validation. Majority (8/11) of the studies did examine reliability.^33–37,40,42,43^ The quality assessments for the articles are illustrated with a heat map in Figure 3.

## Discussion

We aimed to identify and evaluate the quality of survey tools or instruments that have been validated to measure HCW vaccine confidence, hesitancy, or acceptance. We identified 10 articles that developed and conducted validation on a tool to measure HCW vaccine hesitancy or confidence.

The tools developed covered a range of dimensions and topics relating to HCW vaccine confidence using inputs from experts. However, only the three articles on the Pro-VC-Be tool clear lay out the dimension selection process.^33,36,41^ Particularly, for the short-form Pro-VC-Be in which the authors ran a confirmatory factor analysis on all combination of dimensions to determine the most suitable item for each dimension. Others gave some background on the dimension and item development.^34,35,38–40^ Tomietto, et al. used the existing VAX tool, and thus had predefined dimensions for the validation process.^40^ Then Slaunwhite developed the items based on the Theory of Planned Behavior framework and a modified Perceived Behavior Control item.^39^

Despite the variation in reporting of the dimension inclusion, all the studies aimed to examine the main determinants of vaccine confidence and behaviors. All the tools, except versions of Pro-VC-Be, had dimension assessing the HCWs knowledge and/or skills around vaccination. Knowledge and awareness around vaccines has been shown to be associated with positive vaccine attitudes and likelihood to vaccinate.^47^ Dimension on attitude, behavior, and influence allows the tools to assess the 3Cs of vaccine hesitancy: confidence, complacency, and convenience.^11^ Many of the tools also looked at attitudes toward vaccines, such as safety, effectiveness, vaccine usefulness, and moral responsibility, which all have been shown to be important factors influence confidence.^48,49^ A potentially important part of attitude is trust in vaccines or the health system, which five of the studies included as a dimension.^33,36,38,40,41^

The Pro-VC-Be tools were the most well-validated tool identified, which was validated three times in a long-form, short-form and international adaption successfully tested in seven countries and four languages.^33,36,41^ The other tools had less rigorous validation, thus it is important to ensure thorough validation processes in survey development studies. In a scoping review of validated tools to measure vaccine hesitancy from 2010 to 2019, the authors found 26 articles that mostly focused on parent perceptions of routine childhood immunizations and none on validating tools among HCWs.^21^ The studies presented in our systematic review help to fill the gap between the validated tools on HCW vaccine confidence.^21,41^

### Literature gaps

Results show while that there have been efforts to develop and validate tools, there were some key gaps in the literature and limitations with the existing studies. This, despite the fact that, HCW was identified as a core hesitant group during the COVID-19 pandemic.^15^

Furthermore, our systematic review highlighted the limited geographical and income-level scope of existing research. Only one study, conducted in Israel,^37^ took place outside of Europe and North America. Additionally, the data is restricted to high-income countries. This could be due to disease prioritization in the geographical area. However, this limitation hinders the generalizability of the findings and fails to account for the unique challenges and dynamics of vaccine confidence in low- and middle-income settings. A study protocol looking to validate the 5Cs scale among HCWs in South Africa was found, which would help expand the literature scope.^31^ Grjesing et al. explain that the validation of a tool in one region does not mean that it is reliable and valid in another time, culture, and context.^50^ Thus, to ensure comprehensive insights into this global issue, tool validation efforts should extend to other geographic regions.

All except one^43^ of the articles reviewed on tool development and validation for assessing vaccine hesitancy among HCWs were published in the last decade. This temporal gap suggests that while awareness of the importance of understanding HCW hesitancy toward has gained increasing attention, research on validated tools for HCW vaccine hesitancy is still emerging.^21,51^

While the findings from this study revealed that some studies showed good quality and comprehensive validity, others had gaps in the validation processes. Not conducting a criterion validity or having a limited explanation is a prominent challenge encountered during the validation process. The eight studies, that did not conduct a criterion validation, are then most reliant on the content validation and reliability. This means it could be a reliable tool but knowing if the tool is measuring the correct concept is more challenging and the only data presented for validity in these articles is the content or face validations. The lack of criterion validation could be because of lack of existing, widely recognized tools to measure vaccine hesitancy among HCWs, which makes it difficult to assess the criterion validity of new tools.^52,53^ Consequently, there is a need for the establishment of standard reference and more validation of tools specifically for HCWs contexts.^52,53^

Another limitation to the articles included is that several of the studies did not fully elaborate on the effects of the validation processes. The titles, aims, and even parts of methods centered on tool development and validation, while the discussions and conclusions in several of these studies have a primary focus on presenting the survey data.^34,35,37,38,40,43^ Thus, it was difficult for the reader to assess what changes should be made to a tool to improve validation or reliability of the tools. This highlights the importance of refocusing research efforts to place a stronger emphasis on how validation informs tool development and application to other studies.^21^

### Study design limitations

There are a few limitations to our study. First, the initial screening for short list generation was done by one researcher (EG) due to the high volume of searches returned. Second, although we did not have an English language restriction on the search, the diversity of languages meant that for two tools only one research extracted, and quality assessed the data.

### Suggestions for further research

While numerous studies exist that assess vaccine hesitancy in this population, validation processes of tools/instruments to accurately measure vaccine confidence have been overlooked or addressed inadequately.^21,51,54–56^ Our study highlights the necessity for further research specifically dedicated to the validation and reliability of tools designed for assessing vaccine hesitancy among HCWs and considering using existing validated tools, such as the Pro-VC-Be.^33,37,41^ Alternatively, researchers should engage in full validation processes to ensure the reliability and validity of tools designed for specific contexts. If developing a new tool, researchers should consider including dimensions on knowledge, attitudes, trust in system, and vaccination behavior.

Additionally, research efforts should be expanded to encompass low- and middle-income countries is vital to gain a more inclusive understanding of different socio-economic and healthcare contexts that may present unique challenges and require tailored intervention.^57^

## Conclusion

We conclude that the Pro-VC-Be tool as the most useful for future research and can be used as a standard for criterion validation because the Pro-VC-Be underwent a robust validation process.^34^ Additionally, our systematic review emphasizes the critical need for more culturally adapted and standardized tools for assessing vaccine hesitancy among HCWs. Addressing discrepancies in the existing research settings can significantly contribute to the understanding of HCWs vaccine hesitancy and inform targeted interventions and policies in a variety of settings.

## Appendix A

**Table:** Appendix 1. Full search strategy.

Interface: Ovid MEDLINE(R) ALL Date of Search: 8 June 2023 Number of hits: 6,988 Comment: In Ovid, two or more words are automatically searched as phrases; i.e. no quotation marks are needed	Field labels exp/ = exploded MeSH term / = non exploded MeSH term .ti,ab,kf. = title, abstract and author keywords adjx = within x words, regardless of order * = truncation of word for alternate endings
Database(s): **Ovid MEDLINE(R) ALL **1946 to June 07, 2023Search Strategy:	

#	Searches	Results
1	Immunization/	53535
2	Immunization Programs/	12789
3	exp Vaccination/	108774
4	exp Vaccines/	276147
5	(immuni?at* or nonvaccin* or non-immun* or nonimmun* or unimmun* or un-immun* or unvaccin* or vaccin*).ti,ab,kf.	490336
6	or/1-5	566788
7	Anti-Vaccination Movement/	179
8	Patient Acceptance of Health Care/	54724
9	exp Vaccination Refusal/	1640
10	(anti-vaccin* or antivaccin* or anti-vax* or antivax*).ti,ab,kf.	1229
11	((vaccin* or immuni?at*) adj3 (confiden* or delay* or hesitan* or refuse? or refusing or refusal*)).ti,ab,kf.	9543
12	Anxiety/	106052
13	Awareness/	21850
14	Behavior/	30181
15	Choice Behavior/	34903
16	Communication Barriers/	7250
17	Consciousness/	13578
18	Decision Making/	104480
19	Fear/	38267
20	Health Knowledge, Attitudes, Practice/	126411
21	Intention/	16204
22	exp Mandatory Programs/	7181
23	Trust/	12842
24	((immuni?at* or vaccin*) adj3 (accept* or anxi* or attitude* or awareness or barrier* or behavio?r* or belief* or choice* or compulsory or concern* or conscious* or controvers* or critic* or decision-make* or decision-making* or dilemma* or distrust or doubt* or dropout* or enable* or exemption* or fear* or intent* or knowledge or mandatory or misconception* or misinformat* or mistrust* or objection* or objector* or opposition* or perception* or reject* or reluctan* or rumo?r* or trust* or uptake* or willing* or unconscious* or unwilling*)).ti,ab,kf.	28305
25	exp Vaccination/px	2814
26	or/7-25	552350
27	6 and 26	38904
28	exp Health Personnel/	610219
29	((clinical or health or health care or healthcare or medical) adj3 (personnel or professional* or provider* or staff or worker*)).ti,ab,kf.	365983
30	(clinician* or general practitioner* or nurse* or pharmacist* or physician*).ti,ab,kf.	1085664
31	or/28-30	1683215
32	27 and 31	10836
33	Benchmarking/	17206
34	Health Care Surveys/	34008
35	Quality Assurance, Health Care/	56887
36	“Surveys and Questionnaires”/	560894
37	(benchmark* or best practice analy* or feedback* or form? or instrument? or metric? or measure* or nonrespondent? or non-respondent? or questionnair* or respondent* or survey* or tool or tools).ti,ab,kf.	7214411
38	((assessment* or assurance or qualit*) adj3 (care or health care or healthcare)).ti,ab,kf.	126031
39	or/33-38	7430269
40	32 and 39	6988

**Table:** 2. Web of Science Core Collection.

Interface: Clarivate Analytics Editions = A&HCI, ESCI, SCI-EXPANDED, SSCI Date of Search: 8 June 2023 Number of hits: 4,599	Field labels TS/Topic = title, abstract, author keywords and Keywords Plus NEAR/x = within x words, regardless of order * = truncation of word for alternate endingsNote: the *Exact search*-function was used for all the searches

#	Search Query	Results
1	TS=(immuni$at* OR nonvaccin* OR non-immun* OR nonimmun* OR unimmun* OR un-immun* OR unvaccin* OR vaccin*)	513,089
2	TS=(anti-vaccin* OR antivaccin* OR anti-vax* OR antivax*)	1,370
3	TS=((vaccin* OR immuni$at*) NEAR/2 (confiden* OR delay* OR hesitan* OR refuse$ OR refusing OR refusal*))	9,416
4	TS=((immuni$at* OR vaccin*) NEAR/2 (accept* OR anxi* OR attitude* OR awareness OR barrier* OR behavio$r* OR belief* OR choice* OR compulsory OR concern* OR conscious* OR controvers* OR critic* OR decision-make* OR decision-making* OR dilemma* OR distrust OR doubt* OR dropout* OR enable* OR exemption* OR fear* OR intent* OR knowledge OR mandatory OR misconception* OR misinformat* OR mistrust* OR objection* OR objector* OR opposition* OR perception* OR reject* OR reluctan* OR rumo$r* OR trust* OR uptake* OR willing* OR unconscious* OR unwilling*))	27,293
5	#2 OR #3 OR #4	31,767
6	TS=((clinical OR health OR “health care” OR healthcare OR medical) NEAR/3 (personnel OR professional* OR staff OR worker*))	265,043
7	TS=(clinician* OR “general practitioner*” OR nurse* OR pharmacist* OR physician*)	903,018
8	#6 OR #7	1,097,184
9	TS=(benchmark* OR “best practice analy*” OR feedback* OR form$ OR instrument$ OR metric$ OR measure* OR nonrespondent$ OR non-respondent$ OR questionnair* OR respondent* OR survey* OR tool OR tools)	12,756,723
10	TS=((assessment* OR assurance OR qualit*) NEAR/2 (care OR “health care” OR healthcare))	116,891
11	#9 OR #10	12,813,231
12	#1 AND #5 AND #8 AND #11	4,599

**Table:** 3. CABI: CAB Abstracts and Global Health.

Interface: Clarivate Analytics Date of Search: 8 June 2023 Number of hits: 4702	Field labels DE = descriptors TS/Topic = Abstract, BHTD Critical Abstract, Broad Descriptors, CABICODES Names, Descriptors, English Title, Foreign Title, Geographic Location, Identifiers, Organism Descriptors NEAR/x = within x words, regardless of order * = truncation of word for alternate endings Note: the *Exact search*-function was used for all the searches

#	Search Query	Results
1	TS= (immuni$at* OR nonvaccin* OR non-immun* OR nonimmun* OR unimmun* OR un-immun* OR unvaccin* OR vaccin*)	348,475
2	DE = (vaccines OR DNA vaccines OR Haemophilus influenzae vaccines OR acellular vaccines OR autogenous vaccines OR candidate vaccines OR cell culture vaccines OR combined vaccines OR conjugate vaccines OR inactivated vaccines OR live vaccines OR malaria vaccines OR pertussis vaccines OR poliomyelitis vaccines OR polyvalent vaccines OR recombinant vaccines OR synthetic vaccines OR whole cell vaccines)	172,519
3	DE = (immunization programmes) OR DE = (vaccination OR mandatory vaccination OR oral vaccination)	144,164
4	DE = (immunization)	145,047
5	#1 OR #2 OR #3 OR #4	348,475
6	DE = (anxiety)	21,782
7	DE = (awareness)	15,771
8	DE = (behaviour)	321,707
9	DE = (social barriers)	1,333
10	DE = (consciousness OR social consciousness)	1,018
11	DE = (decision making)	55,532
12	DE = (knowledge OR attitudes OR practice)	209,545
13	TS=((immuni$at* OR vaccin*) NEAR/2 (accept* OR anxi* OR attitude* OR awareness OR barrier* OR behavio$r* OR belief* OR choice* OR compulsory OR concern* OR conscious* OR controvers* OR critic* OR decision-make* OR decision-making* OR dilemma* OR distrust OR doubt* OR dropout* OR enable* OR exemption* OR fear* OR intent* OR knowledge OR mandatory OR misconception* OR misinformat* OR mistrust* OR objection* OR objector* OR opposition* OR perception* OR reject* OR reluctan* OR rumo$r* OR trust* OR uptake* OR willing* OR unconscious* OR unwilling*))	17,343
14	TS=(anti-vaccin* OR antivaccin* OR anti-vax* OR antivax*)	658
15	TS=((vaccin* OR immuni$at*) NEAR/2 (confiden* OR delay* OR hesitan* OR refuse$ OR refusing OR refusal*))	5,055
16	#6 OR #7 OR #8 OR #9 OR #10 OR #11 OR #12 OR #13 OR #14 OR #15	571,642
17	DE = (health workers OR health care workers OR careproviders OR community health workers OR dentists OR dietitians OR home health aides OR midwives OR nurses OR nutritionists OR physicians OR traditional birth attendants OR traditional healers)	97,699
18	TS=((clinical OR health OR “health care” OR healthcare OR medical) NEAR/2 (personnel OR professional* OR provider* OR staff OR worker*))	113,305
19	TS=(clinician* OR “general practitioner*” OR nurse* OR pharmacist* OR physician*)	224,720
20	#17 OR #18 OR #19	323,578
21	#5 AND #16 AND #20	6,910
22	DE = (quality assurance)	0
23	DE = (questionnaires)	52,923
24	DE = (surveys)	247,268
25	TS=(benchmark* OR “best practice analy*” OR feedback* OR form$ OR instrument$ OR metric$ OR measure* OR nonrespondent$ OR non-respondent$ OR questionnair* OR respondent* OR survey* OR tool OR tools)	3,551,736
26	TS=((assessment* OR assurance OR qualit*) NEAR/2 (care OR “health care” OR healthcare))	24,641
27	#22 OR #23 OR #24 OR #25 OR #26	3,563,318
28	#27 AND #21	4,719
29	#28 AND CABI: Global Health (CABI Index)	4,702

**Table:** 4. Sociological abstracts.

Interface: ProQuest Date of Search: 8 June 2023 Number of hits: 2,116	Field labels noft = anywhere except full text tiabif = title, abstract, keyword MAINSUBJECT.EXACT = non exploded subject heading MAINSUBJECT.EXACT.EXPLODE = exploded subject heading NEAR/x = within x words, regardless of order * = truncation of word for alternate endings Note: sometimes “quotation marks” are needed for single search terms to avoid automatic term mapping (lemmatization).

S1	title,abstract(immunisat* OR immunizat* OR nonvaccin* OR non-immun* OR nonimmun* OR unimmun* OR un-immun* OR unvaccin* OR vaccin*)	70,099
S2	title,abstract(anti-vaccin* OR antivaccin* OR anti-vax* OR antivax*)	375
S3	title,abstract((vaccin* OR immuni?at*) NEAR/3 (confiden* OR delay* OR hesitan* OR refuse? OR refusing OR refusal*))	2,736
S4	((immunizat* OR immunisat* OR vaccin*) NEAR/3 (accept* OR anxi* OR attitude* OR awareness OR barrier* OR behavior* OR behaviour* OR belief* OR choice* OR compulsory OR concern* OR conscious* OR controvers* OR critic* OR decision-make* OR decision-making* OR dilemma* OR distrust OR doubt* OR dropout* OR enable* OR exemption* OR fear* OR intent* OR knowledge OR mandatory OR misconception* OR misinformat* OR mistrust* OR objection* OR objector* OR opposition* OR perception* OR reject* OR reluctan* OR rumor* OR rumour* OR trust* OR uptake* OR willing* OR unconscious* OR unwilling*))	32,474
S5	S2 OR S3 OR S4	32,885
S6	S1 AND S5	21,904
S7	title,abstract((clinical OR health OR “health care” OR healthcare OR medical) NEAR/3 (personnel OR professional* OR provider* OR staff OR worker*))	79,366
S8	title,abstract(clinician* OR (“general practitioner” OR “general practitioners”) OR nurse* OR pharmacist* OR physician*)	125,061
S9	S7 OR S8	186,043
S10	S6 AND S9	3,453
S11	title,abstract(benchmark* OR “best practice analy*” OR feedback* OR form? OR instrument? OR metric? OR measure* OR nonrespondent? OR non-respondent? OR questionnair* OR respondent* OR survey* OR tool OR tools)	2,483,875
S12	title,abstract((assessment* OR assurance OR qualit*) NEAR/3 (care OR “health care” OR healthcare))	25,040
S13	S11 OR S12	2,494,978
S14	S10 AND S13	2,116

**Table:** 5. Publicly Available Content Database‎.

Interface: ProQuest Date of Search: 8 June 2023 Number of hits: 2,116	Field labels noft = anywhere except full text tiabif = title, abstract, keyword MAINSUBJECT.EXACT = non exploded subject heading MAINSUBJECT.EXACT.EXPLODE = exploded subject heading NEAR/x = within x words, regardless of order * = truncation of word for alternate endings Note: sometimes “quotation marks” are needed for single search terms to avoid automatic term mapping (lemmatization).

S1	title,abstract(immunisat* OR immunizat* OR nonvaccin* OR non-immun* OR nonimmun* OR unimmun* OR un-immun* OR unvaccin* OR vaccin*)	70,099
S2	title,abstract(anti-vaccin* OR antivaccin* OR anti-vax* OR antivax*)	375
S3	title,abstract((vaccin* OR immuni?at*) NEAR/3 (confiden* OR delay* OR hesitan* OR refuse? OR refusing OR refusal*))	2,736
S4	((immunizat* OR immunisat* OR vaccin*) NEAR/3 (accept* OR anxi* OR attitude* OR awareness OR barrier* OR behavior* OR behaviour* OR belief* OR choice* OR compulsory OR concern* OR conscious* OR controvers* OR critic* OR decision-make* OR decision-making* OR dilemma* OR distrust OR doubt* OR dropout* OR enable* OR exemption* OR fear* OR intent* OR knowledge OR mandatory OR misconception* OR misinformat* OR mistrust* OR objection* OR objector* OR opposition* OR perception* OR reject* OR reluctan* OR rumor* OR rumour* OR trust* OR uptake* OR willing* OR unconscious* OR unwilling*))	32,474
S5	S2 OR S3 OR S4	32,885
S6	S1 AND S5	21,904
S7	title,abstract((clinical OR health OR “health care” OR healthcare OR medical) NEAR/3 (personnel OR professional* OR provider* OR staff OR worker*))	79,366
S8	title,abstract(clinician* OR (“general practitioner” OR “general practitioners”) OR nurse* OR pharmacist* OR physician*)	125,061
S9	S7 OR S8	186,043
S10	S6 AND S9	3,453
S11	title,abstract(benchmark* OR “best practice analy*” OR feedback* OR form? OR instrument? OR metric? OR measure* OR nonrespondent? OR non-respondent? OR questionnair* OR respondent* OR survey* OR tool OR tools)	2,483,875
S12	title,abstract((assessment* OR assurance OR qualit*) NEAR/3 (care OR “health care” OR healthcare))	25,040
S13	S11 OR S12	2,494,978
S14	S10 AND S13	2,116

## Appendix B

**Table:** QAVALs quality assessment tool with researcher ratings for included articles.

		Fernandez-Prada et al, 2019	Fernandez-Prada et al, 2016	Garrison et al., 2022		Khamsiy-Farah et al, 2019		Paoli, et al, 2018		Slaunwhite, et al, 2013		Tomietto, et al, 2022		Vergers, et al, 2022		Garrison et al, 2023		Kadir et al, 2021		Zhang et al, 2012	
Item #	Item criteria	EG	EG	EG	KA	EG	KA	EG	KA	EG	KA	EG	KA	EG	KA	EG	KA	EG	KA	EG	KA
1	Was the study design reported?	YES	YES	YES	YES	YES	YES	YES	YES	YES	NO	YES	YES	YES	YES	YES	YES	YES	YES	YES	YES
2	Did the study provide an accurate description of the type of validity tested?	YES	YES	YES	YES	YES	YES	NO	NO	YES	YES	YES	YES	YES	YES	YES	YES	YES	YES	YES	YES
3	Was the study setting and time frame of participant recruitment clearly described?	YES	YES	YES	YES	Other	Other	YES	YES	YES	NO	YES	YES	YES	YES	YES	YES	NO	YES	YES	YES
4	Were the criteria for participant selection clearly described?	YES	YES	YES	YES	YES	NO	YES	YES	YES	NO	YES	NO	YES	Other	YES	Other	YES	YES	YES	YES
5	Were the participants in the study representative of the sample population from which they were recruited?	NO	NO	YES	YES	YES	Other	YES	YES	YES	YES	NO	NO	YES	YES	YES	YES	YES	NO	YES	YES
6	Did the study clearly describe the outcome measures to be validated?	YES	YES	YES	YES	YES	NO	YES	Other	YES	YES	YES	Other	YES	YES	YES	YES	YES	YES	YES	YES
7	Did the study provide a clear description of the procedures for testing validity?	YES	YES	YES	YES	YES	YES	NO	NO	YES	YES	YES	YES	YES	YES	YES	YES	YES	YES	YES	YES
8	Was the testing procedure standardized for all participants?	YES	YES	YES	YES	YES	Other	YES	YES	YES	Other	YES	Other	NO	YES	YES	YES	YES	YES	YES	YES
9	Was a priori sample size calculation performed to ensure that the study had sufficient power?	YES	NO	YES	YES	NO	NO	NO	NO	NO	NO	YES	YES	YES	NO	YES	YES	YES	YES	YES	YES
10	Did the study describe and justify any attrition that may have occurred?	YES	YES	NO	NO	Other	NO	Other	NO	Other	YES	Other	YES	Other	NO	Other	YES	Other	YES	YES	YES
11	Were statistical analyses used to test validity appropriate for the study?	YES	YES	YES	YES	Other	YES	YES	Other	NO	YES	YES	YES	YES	YES	YES	Other	YES	YES	YES	YES
12	When multiple comparisons were performed, were appropriate statistical adjustments used to control for the likelihood of a type 1 error?	YES	YES	Other	Other	YES	NO	YES	Other	YES	NO	YES	NO	YES	NO	YES	YES	YES	YES	NO	NO
13	Did the study identify potential confounding variables and if so, were measures taken to adjust for these confounders?	YES	YES	YES	YES	YES	YES	YES	Other	YES	Other	YES		YES	Other	YES	YES	YES	YES	YES	YES
14	Were primary findings of the study clearly described?	YES	YES	YES	YES	YES	YES	YES	YES	YES	YES	YES	YES	YES	YES	YES	YES	YES	YES	YES	YES
15	Were validity coefficients reported for primary outcomes?	YES	YES	YES	YES	YES	YES	NO	Other	YES		YES	NO	YES		YES	YES	YES	YES	YES	YES
16	For primary outcomes, did the study report standard deviations or confidence intervals for normally distributed data? If non-normally distributed data, did the study report inter-quartile ranges for the main outcomes?	YES	YES	YES	YES	YES	YES	YES	YES	YES	YES	YES	YES	YES	YES	YES	YES	YES	YES	YES	YES
Face/Content Validity																					
17	Was the process of selecting expert panel and their qualifications described?	NO	NO	Other	Other	NO	NO	YES	Other	Other	Other	YES	NO	YES	YES	Other	YES	YES	YES	YES	YES
Criterion Validity																					
18	Did the study provide a rationale for the selection of the reference standard?	NO	Other	YES	YES	Other	Other	Other	Other	Other	Other	Other	Other	YES	YES	YES	YES	Other	NO	Other	Other
19	When the index test was assessed by more than one rater, were the raters blinded to the findings of the other raters?	Other	Other	Other	Other	Other	Other	Other	Other	Other	Other	Other	Other	Other	Other	YES	Other	Other	Other	Other	Other
20	When the index test was assessed by more than one rater, was the inter-rater reliability between raters established and reported?	Other	Other	Other	Other	Other	Other	Other	Other	Other	Other	Other	Other	Other	Other	Other	Other	Other	Other	Other	Other
21	Was the time interval used between administration of reference standard and the test measure appropriate?	YES	Other	YES	YES	Other	Other	Other	Other	Other	Other	Other	Other	YES	Other	Other	Other	Other	Other	Other	Other
Construct Validity (known groups)																					
22	Were subjects in different groups homogenous at baseline? If they weren’t homogenous at baseline, were differences between groups accounted for during the analysis?	Other	YES	Other	Other	Other	Other	Other	Other	Other	Other	Other	Other	Other	YES	Other	YES	Other	Other	Other	Other
Construct Validity (convergent)																					
23	Did the measures used for convergent validity represent a similar construct as the outcome measure of interest?	Other	YES	YES	YES	YES	Other	Other	Other	Other	Other	YES	Other	YES	YES	YES	YES	YES	YES	YES	YES
Construct validity (Divergent)																					
24	Did the measures used for discriminant validity represent a construct different from the outcome measure of interest?	NO	YES	YES	YES	YES	Other	Other	Other	Other	Other	YES	Other	YES	YES	YES	YES	YES	YES	YES	YES
